# The relationship between different levels of facial attractiveness and malocclusion perception: an eye tracking and survey study

**DOI:** 10.1186/s40510-023-00483-2

**Published:** 2023-08-21

**Authors:** Merve Zorlu, Hasan Camcı

**Affiliations:** Department of Orthodontics, Afyonkarahisar Health Sciences University, Güvenevler, İsmet İnönü Cd. No:4, 03030 Afyonkarahisar, Turkey

**Keywords:** Eye tracking, Facial attractiveness, Smile aesthetics, Malocclusion, Gummy smile

## Abstract

**Introduction:**

The aim of this study was to investigate the relationship between levels of facial attractiveness and the perception of different types of malocclusion.

**Methods:**

A preliminary questionnaire was used to assign photographs of three female patients to low, moderate, and high facial attractiveness designations. Seven modified photographs for each smile photograph of each of these three patients were created. The evaluated photographs were as follows: P0: at rest position, P1: ideal smile, P2: − 2-mm (low) smile line, P3: + 4-mm gummy smile, P4: + 6-mm gummy smile, P5: maxillary anterior crowding, P6: median diastema, P7: polydiastema. An eye tracking device and a questionnaire were used to collect data from orthodontists, dentists, orthodontic patients, and laypeople.

**Results:**

Total fixation duration varied depending on the type of malocclusion, the level of facial attraction, and the participants’ occupations. In general, orthodontists and dentists had higher total fixation duration scores than orthodontic patients and laypersons. The maxillary anterior crowding photograph had the lowest visual analysis scale score at each attractiveness level (low, medium, and high). Visual analysis scale scores became similar at each attractiveness level only in the P4 photographs, and thus the difference in facial attractiveness disappeared.

**Conclusion:**

While a worsening of the ideal smile had a smaller impact on aesthetic perceptions in an individual with low facial attractiveness, it had a significant negative impact on a person with high facial attractiveness. Anterior crowding and diastema had a more negative impact on facial attractiveness than low or high smile lines.

## Introduction

Good facial aesthetics have been a desirable physical feature in all societies for centuries [1]. Attractive people are perceived as more socially competent, intelligent, successful, and adorable [2]. As a result, the importance of facial aesthetics is growing by the day.

Facial and dental aesthetic perception are subjective concepts that can differ from person to person or from society to society [3, 4]. The literature is divided on the issue of whether dental attractiveness affects facial attractiveness. According to some researchers, dental aesthetics have an impact on overall facial attractiveness scores. Some studies, however, suggest that other facial structures have a greater influence on facial attractiveness and can suppress smile aesthetics [5, 6].

The evaluation of facial aesthetics is divided into three categories: macroaesthetics, miniaesthetics, and microaesthetics. Macroaesthetics include elements like the ratio of the lips and nose, the vertical and transversal proportions of the face, and chin projections [7]. Miniaesthetics refer to the relationship of the teeth to the face, whereas microaesthetics refer to the relationship of the teeth to each other [8]. Many studies have shown that the eyes are the first area that draws attention when looking at a face, followed by the mouth [9]. However, when factors that negatively affect facial or dental aesthetics are present, attention is drawn to these areas that negatively affect facial harmony. For instance, the eyes are more focused around the mouth when there is a condition present that impairs the aesthetics of a smile [10]. So, can micro or miniaesthetic elements be disregarded if one’s macroaesthetics are excellent? In other words, is there a relationship between the general facial attractiveness level and the perception of the malocclusion type?

Currently, most people with malocclusion seek orthodontic treatment for psychological and social reasons rather than physical and biological ones. Malocclusion, which reduces the aesthetics of smiles, can lead to a variety of psychosocial problems for individuals [11]. When smiling, crowded teeth can significantly reduce one’s facial attractiveness [5, 12, 13]. To the best of our knowledge, no study has been published that investigates the relationship between various malocclusions (e.g., median diastema, crowding, polydiastema, gummy smile, low smile line) and levels of facial attractiveness.

In recent decades, eye tracking systems have been used to assess smile or general facial aesthetics perceptions. The pupil-corneal reflection technique is used in the eye tracking system to record eye movements using special software and equipment. The brain only records information while the eyes focus on a point or area [14]. If a viewer finds a particular area of interest, their gaze will be drawn to it [15]. In comparison with traditional photographic survey studies that evaluate aesthetic perception, the eye tracking system is more advantageous because it can provide more objective findings [16].

The aim of the present study was to examine the relationship between the level of facial attractiveness (low, moderate, and high) and malocclusion perception. Data were collected from four different groups (orthodontists, dentists, patients, and non-professionals) using both questionnaires and an eye tracking device. The data were examined comparatively.

## Materials and methods

The research protocol of this study was approved by the Afyonkarahisar Health Science University Clinical Research Ethics Committee (ID:2022/124). All individuals whose photographs were used in the study, as well as their legal guardians, were asked to sign informed consent forms.

**Fig. 1:**
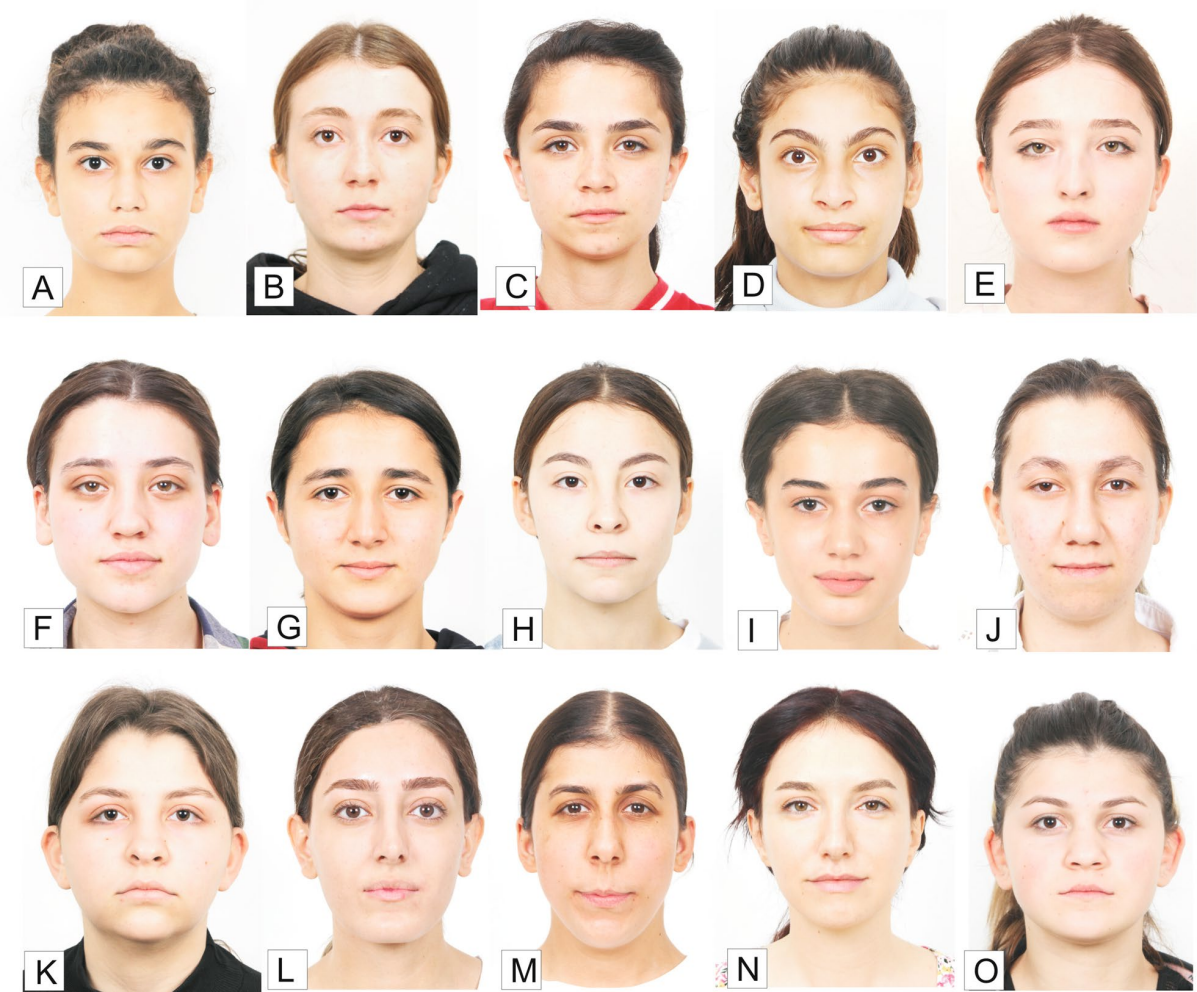
Photographs of the 15 female patients evaluated in the preliminary questionnaire

**Fig. 2:**
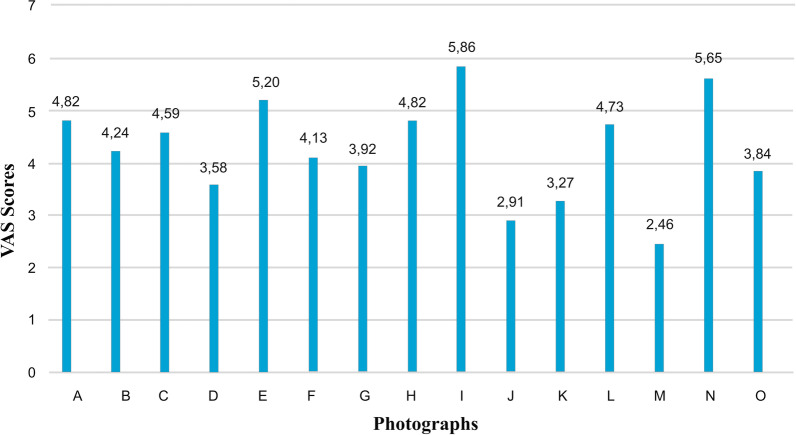
Preliminary VAS score results for the 15 female patients

**Fig. 3:**
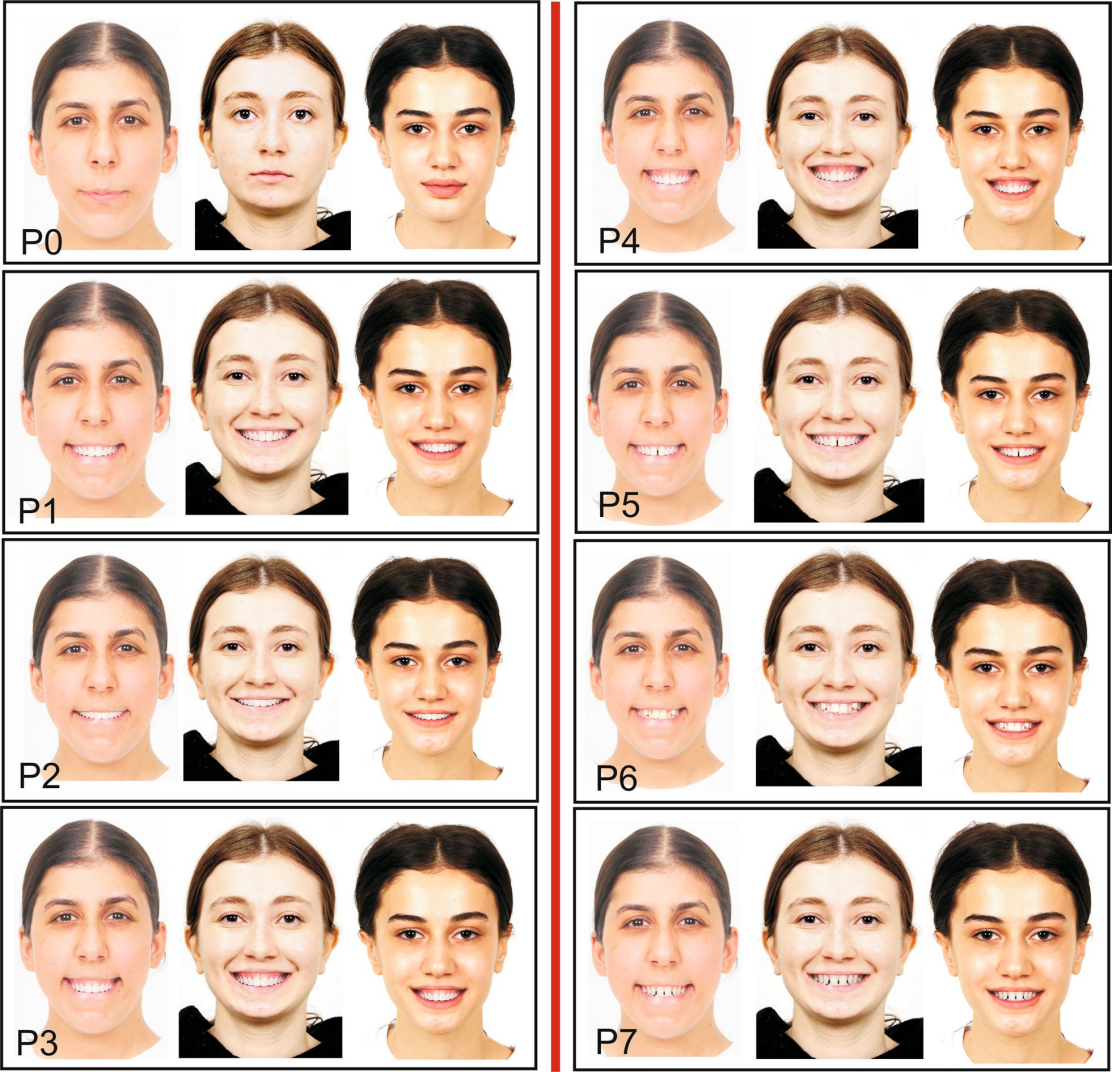
All the resting and modified photographs used in the main survey

A preliminary questionnaire was used to determine photographs of three female patients with low, moderate, and high facial attractiveness. Initial frontal rest photographs of 15 women aged 14–25 were chosen at random from the archive for this purpose (Fig. 1). Individuals with significant facial asymmetry, scars, cleft lips and palates, tattoos, fake eyelashes, or unusual hair styles and colors were excluded from the study. Fifteen photos were randomly organized, and 200 laypeople were asked to rate their attractiveness on a scale of 1 to 10. The raters were not made aware of the project’s goal in order to prevent it from influencing the results. The photographs of the lowest (15th) attractive patient (LAP), the moderate (8th) attractive patient (MAP), and the highest (1st) attractive patient (HAP) were determined based on the results of the preliminary questionnaire (Fig. 2). Seven modified photographs were created using the smile photograph for each of these three patients. The modifications were made using Adobe 2020 Photoshop software. Many different variables have been evaluated in the literature when assessing the relationship between facial attractiveness and smile aesthetics, including varying levels of smile lines, black triangular areas, buccal corridor widths, midline deviations, polydiastema, maxillary anterior crowding, and midline deviation [17–20]. According to Ker et al., a + 3.6-mm gummy smile has a negative impact on facial aesthetics, whereas + 2.1 mm of gingival appearance is considered normal [21]. Therefore, + 4-mm and + 6-mm gummy smile modifications were preferred in the current study. In addition, common malocclusions such as maxillary anterior crowding, median diastema, and polydiastema were included in the study. The modified photographs were as follows: P1: ideal smile, P2: − 2-mm (low) smile line, P3: 4-mm gummy smile, P4: 6-mm gummy smile, P5: maxillary anterior crowding, P6: median diastema, P7: polydiastema. Finally, resting photographs (P0) of each patient were also added. As a result, 24 images in total were assessed for this study (Fig. 3).

The sample size was calculated using G power software. The results of this analysis (alpha = 0.05, effect size = 0.25, 1-B = power 0.80) revealed that at least 180 participants were required. Four different groups were identified: orthodontists, dentists, orthodontic patients, and laypeople. Fifty participants were planned for each group in order to increase the study’s power, and 200 participants in total participated in the investigation. The age range of the participants was between 18 and 50. The exclusion criteria were the presence of a neurological disorder, recent drug or alcohol use that would impair cognitive abilities, any eye or eyelid anomalies, severe visual impairment, or use of photochromic glasses.

**Fig. 4:**
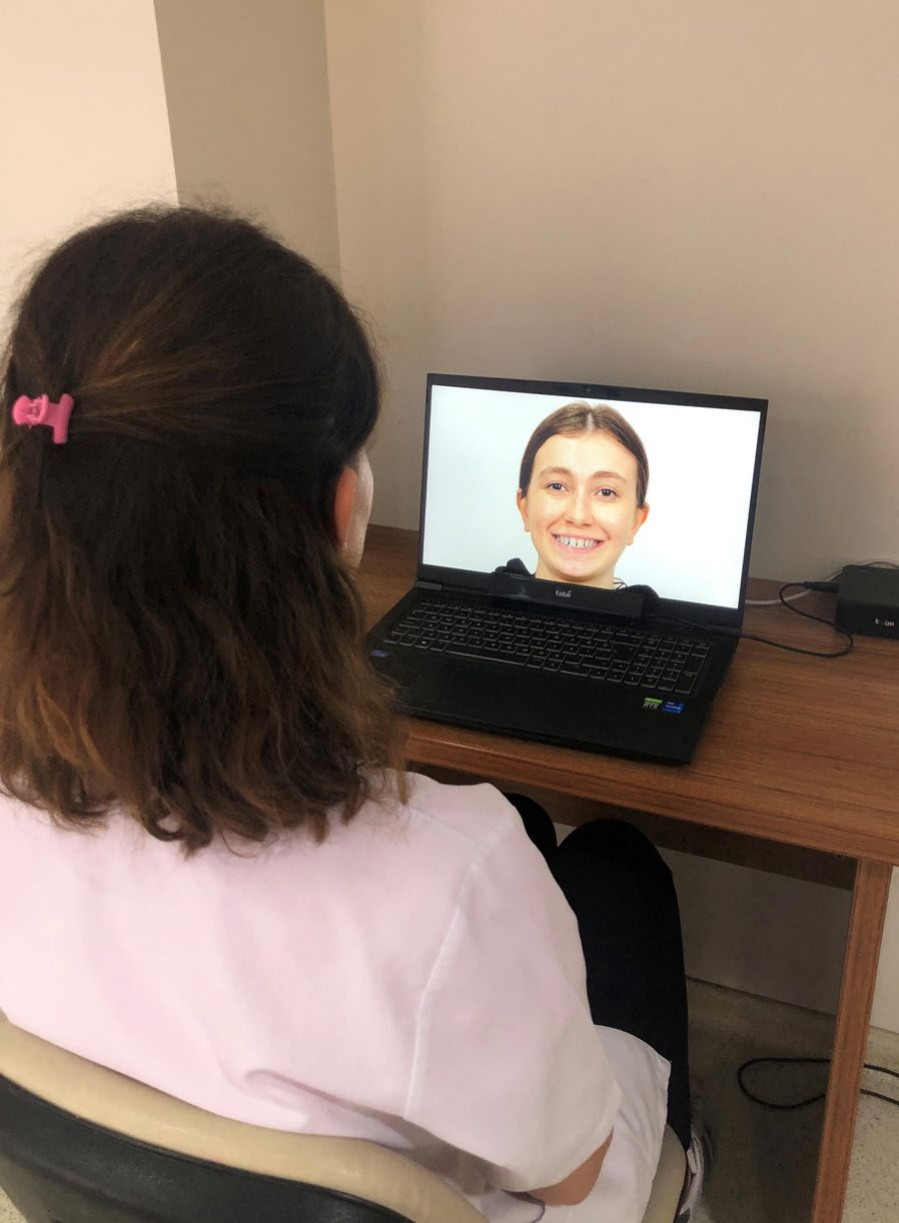
Data collection with eye tracking system

**Fig. 5:**
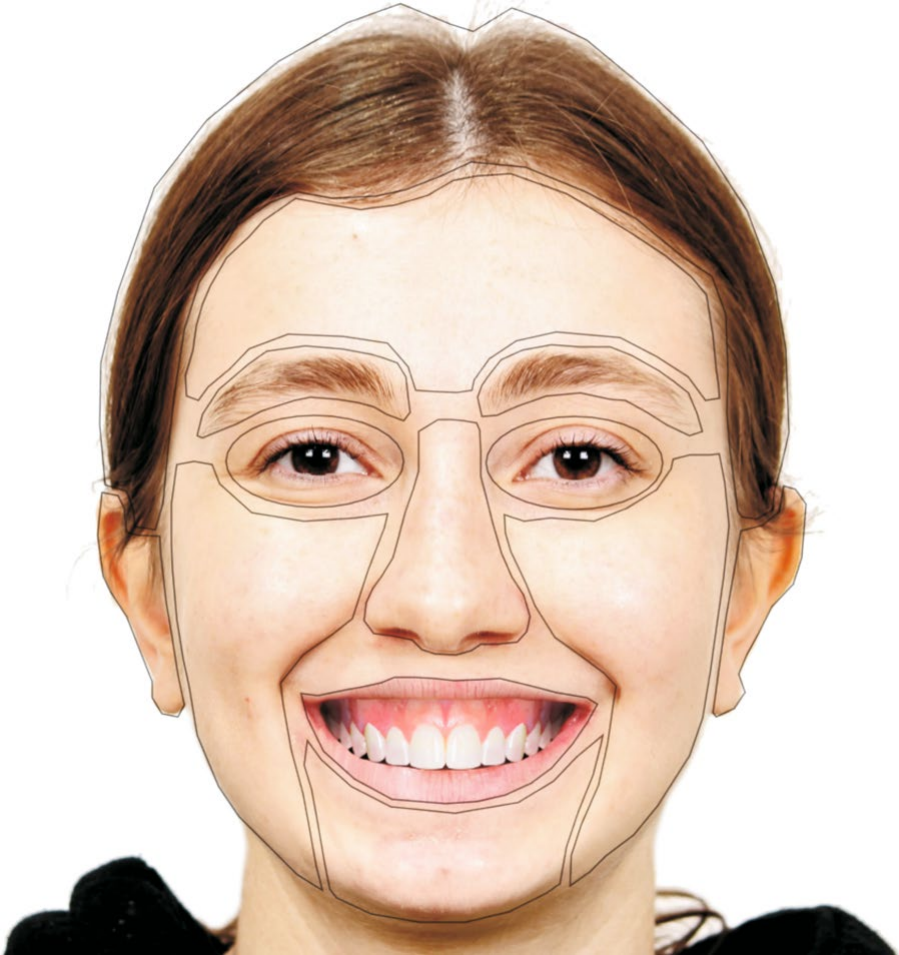
Identified areas of interests

**Fig. 6:**
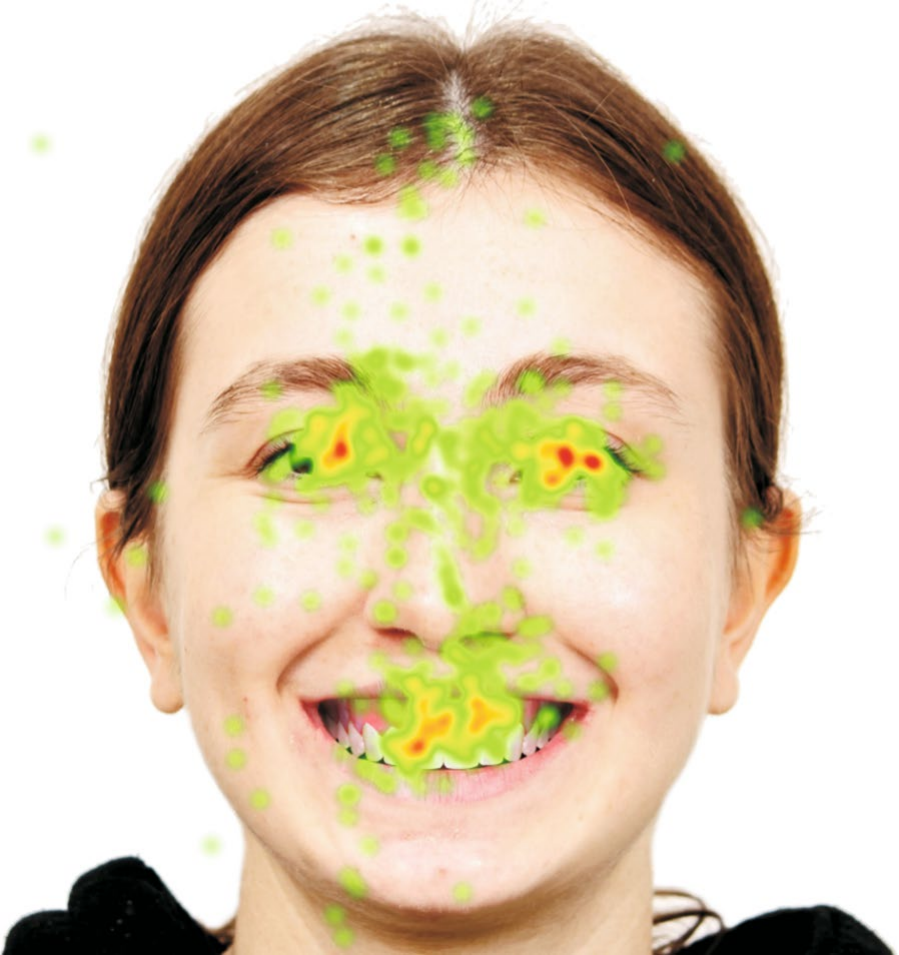
Eye tracking system heatmap results

The Tobii X2-60 Hz (Tobii Technology, Stockholm, Sweden) eye tracking device was used to record the participants’ eye movements. The eye tracking device was placed beneath and in the center of the computer screen, and the distance between the participant and the device was set at 60–65 cm (Fig. 4). Calibration was performed prior to recording each participant’s eye movements in order for the device to function properly. Each participant was then shown the 24 images at random. The display time for each photo was five seconds [16, 22, 23]. The + symbol appeared for one second in the corners or the center of a white blank screen in between each image. As a result, each patient’s eye tracking records were taken in an average of 2.5 min. For each photograph, the mouth area was selected as an area of interest (Fig. 5). Total fixation duration (TFD) parameters related to this region were used in the statistical analysis (Fig. 6).

After the eye tracking recordings, each participant was asked to complete the questionnaire on the same computer. In terms of attractiveness, participants gave a score of 1 to 10 (visual analysis scale, VAS) to each of the 24 randomly placed photographs [24, 25]. The questionnaire was also used to collect demographic data from the participants.

### Statistical analysis

All statistical analyses were performed using the SPSS.25 program. The Kolmogorov–Smirnov test was used to examine the normality assumptions of the continuous variables. The Kruskal–Wallis test was used for intergroup comparisons, and the Bonferroni test was used for post hoc analysis. For intragroup comparisons, the Friedman test was used, and for post hoc analysis, the Bonferroni test was used. The significance value was accepted as *p* < 0.05 in all analyses except Bonferroni-corrected multiple comparisons. Furthermore, Cronbach’s alpha values for VAS and TFD scores were calculated separately.

## Results

The mean age of all participants was 26.6 ± 6.54. The average age of each participant group was as follows: orthodontists 29 ± 4.11, dentists 28.9 ± 4.53, orthodontic patients 20.8 ± 4.31, and laypersons; 27.7 ± 8.40. Cronbach’s alpha was 0.96 for the VAS scores and 0.97 for the TFD scores.

Comparisons of TFD for the LAP photographs are given in Table 1. Orthodontists and dentists generally had higher TFD values than patients and laypersons for all photographs. Between laypeople and patients, there was no statistically significant difference in TFD scores for either the ideal smile photograph or other types of malocclusion. The highest TFD scores for orthodontists were P4 (1.91 ± 1.38), P6 (1.82 ± 1.15), and P5 (1.78 ± 1.26). The P4 image had the highest TFD score (2.29 ± 1.41) for dentists.

**Table 1:** Results of comparing TFD scores for LAP images

	Photographs								*p*
	P0	P1	P2	P3	P4	P5	P6	P7	
Orthodontist	.54 ± .61^A^	1.22 ± 1.08^AC^	1.14 ± 1.03^AB^	1.65 ± 1.13^AB^	1.91 ± 1.38^AC^	1.78 ± 1.26^A^	1.82 ± 1.15^A^	1.66 ± 1.17^AB^	< .001^β^
Dentist	.48 ± .87^AB^	1.61 ± 1.32^A^	1.51 ± 1.47^A^	2.05 ± 1.43^A^	2.29 ± 1.41^A^	1.80 ± 1.44^A^	1.92 ± 1.46^A^	2.06 ± 1.44^A^	< .001^β^
Orthodontic Patient	.19 ± .35^B^	.80 ± 1.26^B^	.96 ± 1.19^B^	1.17 ± 1.51^B^	1.06 ± 1.44^B^	.95 ± 1.22^B^	1.07 ± 1.40^B^	1.16 ± 1.36^B^	< .001^β^
Lay persons	.34 ± .74^B^	.94 ± 1.26^BC^	.86 ± 1.17^B^	1.17 ± 1.31^B^	1.27 ± 1.52^BC^	1.16 ± 1.42^B^	1.24 ± 1.55^B^	1.25 ± 1.58^B^	< .001^β^
*p*	.001^α^	< .001^α^	< .001^α^	< .001^α^	< .001^α^	< .001^α^	< .001^α^	< .001^α^	
Total average	.38 ± .68	1.14 ± 1.26	1.11 ± 1.24	1.50 ± 1.39	1.63 ± 1.50	1.42 ± 1.37	1.51 ± 1.43	1.53 ± 1.42	

In intergroup comparisons, different letters in the same column indicate statistically significant difference

Intragroup comparisons: For Orthodontists: 0 < 1, 2, 3, 4, 5, 6 ve 7; 1 < 4, 5, 6; 2 < 3, 4, 5, 6 ve 7. Dentists: 0 < 1, 2, 3, 4, 5, 6 ve 7; 1 < 4; 2 < 3, 4, 7. Orthodontic patients: 0 < 1, 2, 3, 4, 5, 6 ve 7. Lay persons: 0 < 1, 2, 3, 4, 5, 6 ve 7

P0, Resting; P1, Ideal smile; P2, − 2 mm low smile line; P3, + 4 mm gingival smile; P4, + 6 mm gingival smile; P5, Maxillary anterior crowding; P6, Median diastema; P7, Polydiastema

^α^Kruskal–Wallis test, *p* value < 0.05

^β^Friedman test, *p* value < 0.017

The VAS values for the LAP photographs are shown in Table 2. There was no statistically significant difference between the groups when the P5, P6, and P7 VAS scores were compared. P2, P3, and P4 scores were higher for patients and laypeople than P5, P6, and P7. In other words, anterior crowding and diastema had a greater negative impact on facial aesthetics than low or high smile lines. While the ideal smile photographs received the highest scores in all the groups, the VAS scores decreased in the presence of malocclusion.

**Table 2:** Results of comparing VAS scores for LAP images

	Photographs								*p*
	P0	P1	P2	P3	P4	P5	P6	P7	
Orthodontist	2.62 ± 1.44^A^	3.80 ± 1.70^A^	3.04 ± 1.47^A^	2.56 ± 1.31^A^	2.24 ± 1.32^A^	2.22 ± 1.45^A^	2.44 ± 1.33^A^	2.18 ± 1.30^A^	< .001^β^
Dentist	3.08 ± 1.35^AB^	4.46 ± 1.93^AB^	3.90 ± 1.71^AB^	3.00 ± 1.63^AB^	2.86 ± 1.65^AB^	2.52 ± 1.30^A^	2.72 ± 1.51^A^	2.62 ± 1.26^A^	< .001^β^
Orthodontic patient	3.42 ± 1.62^B^	5.06 ± 2.56^AB^	4.18 ± 2.14^B^	4.10 ± 2.44^B^	3.80 ± 2.42^B^	2.38 ± 1.52^A^	2.74 ± 1.47^A^	2.54 ± 1.45^A^	< .001^β^
Lay persons	3.48 ± 1.76^B^	5.26 ± 2.41^B^	4.64 ± 2.07^B^	4.20 ± 2.47^B^	4.02 ± 2.55^B^	2.60 ± 1.54^A^	2.96 ± 1.65^A^	2.80 ± 1.86^A^	< .001^β^
*p*	.018^α^	.007^α^	< .001^α^	.001^α^	< .001^α^	.389^α^	.496^α^	.243^α^	
Total average	3.15 ± 1.57	4.64 ± 2.23	3.94 ± 1.94	3.46 ± 2.13	3.23 ± 2.15	2.43 ± 1.45	2.71 ± 1.49	2.53 ± 1.49	

In intergroup comparisons, different letters in the same column indicate statistically significant difference

Intragroup comparisons: For Orthodontists: 0 < 1; 1 > 2, 3, 4, 5, 6 ve 7; 2 > 4, 5, 6, 7. Dentists: 0 < 1, 2; 1 > 2, 3, 4, 5, 6, 7; 2 > 3, 4, 5, 6, 7. Orthodontic patients: 0 < 1, 2; 1 > 2, 3, 4, 5, 6 ve 7. 2 > 5, 6, 7; 3 > 5, 6, 7. 4 > 5, 6, 7. Lay persons: 0 < 1, 2; 0 > 5, 7. 1 > 3, 4, 5, 6 ve 7; 2 > 5, 6, 7; 3 > 5, 6, 7; 4 > 5, 6, 7

P0, Resting; P1, Ideal smile; P2, − 2 mm low smile line; P3, + 4 mm gingival smile; P4, + 6 mm gingival smile; P5, Maxillary anterior crowding; P6, Median diastema; P7, Polydiastema

^α^Kruskal–Wallis test, *p* value < 0.05

^β^Friedman test, *p* value < 0.017

Comparisons of the TFD scores of the MAP photographs are given in Table 3. The highest TFD value for orthodontists was for P5 (2.03 ± 1.29), but there was no statistically significant difference between P3, P4, P5, P6, and P7. P3, P4, P5, and P6 had the highest TFD values in the dentist group. The TFD values of patients and laypeople were generally found to be lower than those of dentists and orthodontists. The P4 photograph (1.51 ± 1.57) had the highest TFD value in the patient group. In the group of laypeople, there was no statistically significant difference between malocclusion type and the ideal smile in terms of TFD value.

**Table 3:** Results of comparing TFD scores for MAP images

	Photographs								*p*
	P0	P1	P2	P3	P4	P5	P6	P7	
Orthodontist	.35 ± .48^A^	1.44 ± 1.07^A^	1.50 ± 1.20^A^	1.83 ± 1.18^A^	1.73 ± 1.15^AB^	2.03 ± 1.29^AB^	1.60 ± 1.15^AB^	1.63 ± 1.18^AB^	< .001^β^
Dentist	.31 ± .51^AB^	1.71 ± 1.43^A^	1.74 ± 1.39^A^	2.26 ± 1.51^A^	2.28 ± 1.38^A^	2.27 ± 1.55^A^	2.17 ± 1.52^A^	1.82 ± 1.40^A^	< .001^β^
Orthodontic Patient	.13 ± .27^B^	1.15 ± 1.44^AB^	.92 ± 1.43^B^	.93 ± 1.35^B^	1.51 ± 1.57^AB^	1.23 ± 1.51^B^	1.24 ± 1.49^B^	1.18 ± 1.32^AB^	< .001^β^
Lay persons	.18 ± .35^AB^	.87 ± 1.21^B^	.94 ± 1.17^B^	1.40 ± 1.58^AB^	1.43 ± 1.62^B^	1.30 ± 1.57^B^	1.17 ± 1.46^B^	1.13 ± 1.33^B^	< .001^β^
*p*	.021^α^	< .001^α^	< .001^α^	< .001^α^	.003^α^	< .001^α^	< .001^α^	.007^α^	
Total average	.24 ± .42	1.29 ± 1.32	1.27 ± 1.33	1.60 ± 1.48	1.73 ± 1.46	1.70 ± 1.53	1.54 ± 1.45	1.44 ± 1.33	

In intergroup comparisons, different letters in the same column indicate statistically significant difference

Intragroup comparisons: For Orthodontists: 0 < 1, 2, 3, 4, 5, 6 ve 7; 1 < 5; 2 < 5. Dentists: 0 < 1, 2, 3, 4, 5, 6 ve 7; 1 < 3, 4, 5 ve 6; 2 < 3, 4, 5 ve 6. Orthodontic patients: 0 < 1, 2, 3, 4, 5, 6 ve 7; 2 < 4; 3 < 4. Lay persons: 0 < 1, 2, 3, 4, 5, 6 ve 7

P0, Resting; P1, Ideal smile; P2, − 2 mm low smile line; P3, + 4 mm gingival smile; P4, + 6 mm gingival smile; P5, Maxillary anterior crowding; P6, Median diastema; P7, Polydiastema

^α^Kruskal–Wallis test, *p* value < 0.05

^β^Friedman test, *p* value < 0.017

The VAS values for the MAP photographs are shown in Table 4. In the intergroup comparison of VAS scores, only the P3 and P4 photographs showed a statistically significant difference. P4, P5, P6, and P7 had the lowest VAS scores for orthodontists. P4, P5, and P6 in the dentist group received the lowest scores. P5 had the lowest VAS score for both patients and laypersons. In other words, anterior crowding and diastema affected facial attractiveness more negatively than low or high smile lines. Ideal smile photos received the highest scores in all groups, and there was no statistically significant decrease in VAS scores due to a low smile line. The presence of a 6-mm gummy smile (P4), on the other hand, had a negative impact on the VAS score.

**Table 4:** Results of comparing VAS scores for MAP images

	Photographs								*p* value
	P0	P1	P2	P3	P4	P5	P6	P7	
Orthodontist	4.48 ± 1.43^A^	5.98 ± 1.72^A^	5.00 ± 1.44^A^	4.08 ± 1.76^A^	3.02 ± 1.42^A^	3.14 ± 1.67^A^	3.24 ± 1.64^A^	3.00 ± 1.55^A^	< .001^β^
Dentist	4.64 ± 1.52^A^	6.30 ± 1.75^A^	5.68 ± 2.08^A^	4.12 ± 1.78^A^	3.12 ± 1.52^A^	3.00 ± 1.46^A^	3.18 ± 1.38^A^	3.46 ± 1.42^A^	< .001^β^
Orthodontic Patient	4.78 ± 1.85^A^	6.60 ± 2.37^A^	6.00 ± 2.58^A^	5.16 ± 2.33^AB^	4.14 ± 2.37^A^	2.68 ± 1.78^A^	3.32 ± 1.88^A^	3.22 ± 1.82^A^	< .001^β^
Lay persons	4.68 ± 1.95^A^	6.80 ± 2.22^A^	6.08 ± 2.41^A^	5.70 ± 2.48^B^	4.30 ± 2.53^A^	3.06 ± 1.98^A^	3.40 ± 2.00^A^	3.34 ± 1.87^A^	< .001^β^
*p* value	.892^α^	.069^α^	.037^α^	.001^α^	.018^α^	.366^α^	.977^α^	.451^α^	
Total average	4.64 ± 1.69	6.42 ± 2.04	5.69 ± 2.19	4.76 ± 2.21	3.64 ± 2.08	2.97 ± 1.72	3.28 ± 1.72	3.25 ± 1.67	

In intergroup comparisons, different letters in the same column indicate statistically significant difference

Intragroup comparisons: For Orthodontists: 0 < 1, 0 > 4, 5, 6 ve 7; 1 > 2, 3, 4, 5, 6 ve 7; 2 > 4, 5, 6 ve 7; 3 > 4, 5, 6 ve 7. Dentists: 0 < 1, 2, 0 > 4, 5, 6 ve 7; 1 > 3, 4, 5, 6 ve 7; 2 > 3, 4, 5, 6 ve 7; 3 > 4, 5 ve 6. Orthodontic patients: 0 < 1, 2; 0 > 5, 6 ve 7; 1 > 3, 4, 5, 6 ve 7; 2 > 4, 5, 6 ve 7; 3 > 4, 5, 6 ve 7; 4 > 5; 5 < 6. Lay persons: 0 < 1, 2; 0 > 5,6, 7; 1 > 3, 4, 5, 6 ve 7; 2 > 4, 5, 6 ve 7; 3 > 4, 5, 6 ve 7; 4 > 5,6 ve 7

P0, Resting; P1, Ideal smile; P2, − 2 mm low smile line; P3, + 4 mm gingival smile; P4, + 6 mm gingival smile; P5, Maxillary anterior crowding; P6, Median diastema; P7, Polydiastema

^α^Kruskal–Wallis test, *p* value < 0.05

^β^Friedman test, *p* value < 0.017

Comparisons of the TFD values of HAP photographs are given in Table 5. For orthodontists and laypersons, there were no significant differences in the TFD scores of the ideal smile and other malocclusions photographs. The TFD values were generally higher in the orthodontist and dentist groups than in the patient and layperson groups. The P5 photograph received the highest TFD score in the patient group.

**Table 5:** Results of comparing TFD scores for HAP images

	Photographs								*p* value
	P0	P1	P2	P3	P4	P5	P6	P7	
Orthodontist	.54 ± .70^A^	1.54 ± 1.12^A^	1.36 ± 1.16^A^	1.50 ± 1.12^AB^	1.79 ± 1.33^AC^	1.76 ± 1.27^AB^	1.73 ± 1.18^AB^	1.74 ± 1.24^AB^	< .001^β^
Dentist	.58 ± .80^A^	1.79 ± 1.42^A^	1.33 ± 1.17^A^	1.89 ± 1.31^A^	2.22 ± 1.48^A^	2.20 ± 1.42^A^	2.27 ± 1.51^A^	1.98 ± 1.42^A^	< .001^β^
Orthodontic Patient	.30 ± .66^A^	.98 ± 1.46^B^	.66 ± .96^B^	1.12 ± 1.48^B^	1.34 ± 1.44^BC^	1.36 ± 1.51^B^	1.29 ± 1.51^B^	1.20 ± 1.50^B^	< .001^β^
Lay persons	.27 ± .53^A^	.95 ± 1.45^B^	.88 ± 1.21^AB^	1.10 ± 1.34^B^	1.18 ± 1.35^B^	1.32 ± 1.52^B^	1.19 ± 1.27^B^	1.07 ± 1.39^B^	< .001^β^
*p* value	.005^α^	< .001^α^	< .001^α^	.001^α^	.001^α^	.001^α^	< .001^α^	< .001^α^	
Total average	.42 ± .68	1.31 ± 1.40	1.05 ± 1.15	1.40 ± 1.34	1.63 ± 1.44	1.66 ± 1.46	1.62 ± 1.43	1.49 ± 1.43	

In intergroup comparisons, different letters in the same column indicate statistically significant difference

Intragroup comparisons: For Orthodontists: 0 < 1, 2, 3, 4, 5, 6 ve 7. Dentists: 0 < 1, 2, 3, 4, 5, 6 ve 7; 2 < 3, 4, 5, 6 ve 7. Orthodontic patients: 0 < 1, 2, 3, 4, 5, 6 ve 7; 2 < 4 ve 5. Lay persons: 0 < 1, 2, 3, 4, 5, 6 ve 7

P0, Resting; P1, Ideal smile; P2, − 2 mm low smile line; P3, + 4 mm gingival smile; P4, + 6 mm gingival smile; P5, Maxillary anterior crowding; P6, Median diastema; P7, Polydiastema

^α^Kruskal–Wallis test, *p* value < 0.05

^β^Friedman test, *p* value < 0.017

The VAS values for the HAP photographs are shown in Table 6. In the intergroup comparison, there was no statistically significant difference in the VAS scores for P5, P6, and P7. For the orthodontist, patient, and layperson groups, there was no statistically significant difference between the ideal smile and other malocclusion types in terms of VAS scores. P4, P5, P6, and P7 photographs had the lowest VAS scores in the orthodontist group. P4, P5, P6, and P7 received the lowest scores in the dentist group. P5 had the lowest VAS score for both patients and laypeople. Ideal smile photos received the highest scores in all groups, and there was no statistically significant decrease in VAS scores due to a low smile line. The presence of a 6-mm gummy smile (P4), on the other hand, had a negative impact on the VAS score.

**Table 6:** Results of comparing VAS scores for HAP images

	Photographs								*p* value
	P0	P1	P2	P3	P4	P5	P6	P7	
Orthodontist	5.92 ± 1.56^A^	6.22 ± 1.98^A^	6.08 ± 1.59^A^	3.92 ± 1.84^A^	2.60 ± 1.70^A^	3.54 ± 1.75^A^	3.26 ± 1.58^A^	3.04 ± 1.62^A^	< .001^β^
Dentist	6.28 ± 1.75^AB^	6.26 ± 1.91^A^	6.72 ± 1.84^AC^	4.24 ± 2.05^A^	2.72 ± 1.50^A^	3.44 ± 1.47^A^	3.40 ± 1.46^A^	3.44 ± 1.79^A^	< .001^β^
Orthodontic Patient	6.86 ± 1.76^B^	7.10 ± 2.06^AB^	7.46 ± 2.12^BC^	5.84 ± 2.58^B^	4.10 ± 2.31^B^	2.98 ± 1.58^A^	3.40 ± 1.97^A^	3.50 ± 1.88^A^	< .001^β^
Lay persons	6.70 ± 1.78^AB^	7.28 ± 1.99^B^	7.62 ± 1.92^B^	5.78 ± 2.76^B^	4.18 ± 2.50^B^	3.16 ± 1.78^A^	3.32 ± 1.67^A^	3.60 ± 1.96^A^	< .001^β^
*p* value	.029^α^	.006^α^	< .001^α^	.001^α^	< .001^α^	.309^α^	.961^α^	.475^α^	
Total average	6.44 ± 1.74	6.71 ± 2.03	6.97 ± 1.96	4.94 ± 2.47	3.40 ± 2.15	3.28 ± 1.65	3.34 ± 1.66	3.39 ± 1.81	

In intergroup comparisons, different letters in the same column indicate statistically significant difference

Intragroup comparisons: For Orthodontists: 0 > 4, 5, 6 ve 7; 1 > 3, 4, 5, 6 ve 7; 2 > 3, 4, 5, 6, 7; 3 > 4, 7; 4 < 5. Dentists: 0 > 3, 4, 5, 6 ve 7; 1 > 3, 4, 5, 6, 7; 2 > 3, 4, 5, 6 ve 7; 3 > 4 ve 6; 4 < 5, 6 ve 7. Orthodontic patients: 0 > 3, 4, 5, 6 ve 7; 1 > 3, 4, 5, 6, 7; 2 > 3, 4, 5, 6, 7; 3 > 4, 5, 6 ve 7. Lay persons: 0 > 4, 5, 6 ve 7; 1 > 3, 4, 5, 6, 7. 2 > 3, 4, 5, 6, 7; 3 > 4, 5, 6 ve 7

P0, Resting; P1, Ideal smile; P2, − 2 mm low smile line; P3, + 4 mm gingival smile; P4, + 6 mm gingival smile; P5, Maxillary anterior crowding; P6, Median diastema; P7, Polydiastema

^α^Kruskal–Wallis test, *p* value < 0.05

^β^Friedman test, *p* value < 0.017

The general VAS score averages for all participants are given in Table 7. Differences in LAP and MAP VAS scores decreased in the presence of an ideal smile (P1), + 4-mm gingival smile (P3), maxillary anterior crowding (P5), and diastema (P6 and P7). The maxillary anterior crowding photograph had the lowest VAS score at each attractiveness level (LAP, MAP, and HAP). VAS scores became similar at each attractiveness level only in P4 (6-mm gummy smile), and thus the difference in facial attractiveness disappeared.

**Table 7:** Comparison of total scores of all participants for LAP, MAP and HAP photographs

	Photographs							
	P0	P1	P2	P3	P4	P5	P6	P7
LAP	3.15 ± 1.57^A^	4.64 ± 2.23^A^	3.94 ± 1.94^A^	3.46 ± 2.13^A^	3.23 ± 2.15^A^	2.43 ± 1.45^A^	2.71 ± 1.49^A^	2.53 ± 1.49^A^
MAP	4.64 ± 1.69^B^	6.42 ± 2.04^B^	5.69 ± 2.19^B^	4.76 ± 2.21^B^	3.64 ± 2.08^A^	2.97 ± 1.72^B^	3.28 ± 1.72^B^	3.25 ± 1.67^B^
HAP	6.44 ± 1.74^C^	6.71 ± 2.03^B^	6.97 ± 1.96^C^	4.94 ± 2.47^B^	3.40 ± 2.15^A^	3.28 ± 1.65^B^	3.34 ± 1.66^B^	3.39 ± 1.81^B^
*p* value	.001	.001	.001	.001	.149	.001	.001	.001

Kruskal–Wallis test, *p* value < 0.05

In intergroup comparisons, different letters in the same column indicate statistically significant difference

P0, Resting; P1, Ideal smile; P2, − 2 mm low smile line; P3, + 4 mm gingival smile; P4, + 6 mm gingival smile; P5, Maxillary anterior crowding; P6, Median diastema; P7, Polydiastema

## Discussion

There are numerous studies in the orthodontics literature that evaluate facial attractiveness and smile aesthetics. However, there are limited studies evaluating the relationship between facial attractiveness and malocclusion [10, 26, 27]. Generally, a VAS scale is used in studies that assess the aesthetics of the smile and face [18, 28]. There are multiple factors that influence smile aesthetics (e.g., gingival level, tooth axis, lower lip curvature) and multiple factors that affect facial aesthetics (e.g., eye, nose, face shape) [29, 30]. In the current study, in addition to a VAS scale, an eye tracking device was used to determine whether the variables were observed and for how long the variables were focused on. Thus, the relatively subjective VAS score was supported by more objective eye tracking data.

Previous similar studies have used eye tracking devices to investigate the effects of different buccal corridor widths, midline deviations, gingival appearances, and median diastema widths on facial attractiveness [19, 20]. The current study compared the ideal smile to common malocclusions like a gummy smile (− 2, 4, and 6 mm), maxillary anterior crowding, median diastema, and polydiastema. In addition, resting photographs were included in the study, and they were used to investigate how ideal or poor smile aesthetics affect general facial attractiveness. These modifications were assessed by comparing patients with three different levels of facial attractiveness (low, moderate, high) rather than based on a single patient’s frontal photograph. Thus, whether there is a relationship between the level of facial attractiveness and types of malocclusion was investigated.

Similar studies have revealed that the ages of the evaluated persons and the participants may have an impact on the findings [31, 32]. However, some researchers claim that age has no impact on the results [33, 34]. Younger participants, according to Johnston et al., are more critical in their assessments [35]. Facial photographs of adolescents or young adults were modified for previous studies [9]. As a result, photographs of people aged 14 to 25 were preferred in the current study.

The literature is divided on the effect of gender on aesthetic evaluation results [36]. In a study by Cross and Cross, women rated a female face more positively than men did; however, there was no difference between the genders when rating the male face [33]. Some studies claim that gender has no effect on the outcomes of aesthetic perceptions [37–39]. In the current study, only photographs of female patients were evaluated because there was too much data already available. However, the gender distribution of the total participants was equal (100 female and 100 male).

Smile lines were altered by Sriphadungporn and Chamnannidiadha between − 4 and + 6 mm, and by Ioi et al. between − 5 and + 5 mm [40, 41]. According to Ker et al., a 2.1-mm gingival display was considered aesthetic, whereas a 3.6-mm gingival appearance had a negative impact on smile aesthetics [21]. Kokich et al. reported that dentists and laypeople tolerated gingival appearance until the gingival smile line was + 4 mm [42]. In terms of smile aesthetics, a gingival appearance of 1–2 mm is considered normal. Therefore, + 2-mm gingival smile modifications were not used in the current study, and + 4-mm and + 6-mm modifications were preferred.

Facial and smile attractiveness are subjective concepts [3, 4]. There is no agreement in the literature that good dental aesthetics enhance facial attractiveness. Some research suggests that dental aesthetics contribute to overall facial attractiveness. However, some studies claim that the attractiveness of other facial structures (e.g., eyes, nose) is more important [5, 6, 28]. Havens et al. reported that correcting malocclusion (making a smile closer to ideal) increases overall facial attractiveness [43]. Tatarunaite et al. reported that the ideal smile does not improve general facial attractiveness [28]. The authors of the current study suggest that previous studies’ evaluations of patients with different degrees of facial attractiveness may be one factor contributing to these contradictory results. Based on the results of the current study, the ideal smile enhanced facial beauty in individuals with low or medium levels of facial attractiveness but did not significantly enhance facial attractiveness in people with high levels of attractiveness.

Gasparello et al. reported that there was no statistically significant difference in total fixation time between IOTN 1 (Index of Treatment Need) (close to ideal smile), IOTN 5 (malocclusion with median diastema), and IOTN 8 (malocclusion with maxillary anterior crowding) photographs. However, in this study, IOTN 1 had the highest VAS scores [22]. Similarly, in our study, there was no significant difference between the ideal smile, maxillary anterior crowding, and median diastema photographs in terms of total fixation time in the layperson and orthodontic patient groups. In addition, it was observed that TFD was affected by occupation type and level of facial attractiveness.

Using an eye tracking system, Richards et al. and Baker et al. investigated the relationship between three types of malocclusion and three levels of facial attractiveness. In both studies, regardless of facial attractiveness, the focus on the mouth region increased as the severity of the malocclusion increased [10, 26]. Johnson et al. reported similar results [27]. In the current study, TFD was found to be influenced not only by the type of malocclusion, but also by the level of facial attractiveness and participants’ occupations. Orthodontists were found to be more attentive to the maxillary anterior crowding image than dentists in a study by Oliveira et al. [44]. However, in the current study, no significant difference was found between dentists and orthodontists in terms of TFD in any type of malocclusion, including crowding.

According to Tanaka et al., TFD increased as diastema width increased, but TFD was unaffected by participants’ occupations [20]. In our study, it was found that regardless of the degree of facial attractiveness, the TFD scores of orthodontists and dentists were generally higher for the median diastema and polydiastema photographs compared to orthodontic patients and laypersons. The photograph with a + 6-mm smile line had the highest TFD value in Çelikdelen ve Bıçakçı's study among smile lines ranging from − 4 mm to + 6 mm [19]. There was no statistically significant difference between the TFD scores of the + 4- and + 6-mm gummy smile photographs and the ideal smile photographs for laypeople in our study, regardless of the facial attractiveness levels of the photographs. Prasad et al. claimed that maxillary anterior crowding and median diastema affect facial attractiveness more negatively than gummy smiles [45]. There was a statistically significant difference between the VAS scores of the ideal smile photos in the current study, but not between the VAS scores of the + 6-mm gummy smile photos. In other words, the presence of a + 6-mm gummy smile eliminated the difference in facial attractiveness level that existed at the beginning. Furthermore, in our study, the maxillary anterior crowding photo received the lowest scores at each attractiveness level for all groups. Soh et al. found that laypeople scored maxillary anterior crowding lower than orthodontists [46]. In the current study, no statistically significant difference was found between orthodontists and laypersons in photographs of maxillary anterior crowding, regardless of the level of facial attractiveness. Interestingly, in a survey study conducted in Japan, orthodontic patients and laypeople found people with anterior crowding to be attractive [18]. This result demonstrates how social differences can influence perceptions of attractiveness.

There could be a number of reasons why studies on smile aesthetics and facial attractiveness produce differing results. Factors can include malocclusion type and severity, as well as participants’ ethnicities, age ranges, and genders [47]. The most crucial aspect is that aesthetic perception is a personal experience that differs from person to person. The photographs used in the current study were two-dimensional, static images. Although many studies in the literature report that static recordings are a valid method, using three-dimensional dynamic video recordings could have allowed for more accurate assessments. In addition, we excluded from the study one parameter that has a general impact on smile attractiveness: the vertical asymmetry of the smile line. For example, the impact of such asymmetry on smile aesthetics may be greater in subjects who have more gingival exposure than in subjects who have no gingival exposure. The evaluation of only female subjects’ photos in the current study is another limitation. It should also be noted that the photographs were modified artificially. There is a need for additional research in various geographic locations with more participants in order to generalize the findings of the current study.

## Conclusion

- While an ideal smile increased facial beauty in individuals with low and moderate facial attractiveness, it did not make a significant contribution to an individual with high facial attractiveness.
- Orthodontists and dentists in general spent more time focusing on the oral region than patients and laypersons.
- Anterior crowding and diastema had a more negative impact on facial attractiveness than low or high smile lines.
- A low smile line did not reduce facial attractiveness in general.
- While a worsening of the ideal smile had a smaller impact on aesthetic perception in an individual with low facial attractiveness, it had a significant negative impact on a person with high facial attractiveness.

## Data Availability

Data and materials are available at the Orthodontic Department in the Faculty of Dentistry, Afyonkarahisar Health Science University.
